# Left atrial strain rate during atrial contraction predicts raised pulmonary capillary wedge pressure: evidence for left atrio-ventricular interaction

**DOI:** 10.1007/s10554-020-02126-7

**Published:** 2021-01-03

**Authors:** Per Lindqvist, Michael Henein

**Affiliations:** 1 Department of Surgical and Perioperative Sciences and Institute of Public Health and Clinical Medicine, Umeå University, Umeå, Sweden; 2 Institute of Public Health and Clinical Medicine, Umeå University, Umeå, Sweden

**Keywords:** Left atrium, Strain, Strain rate, Pulmonary capillary wedge pressure, Echocardiography, Right heart catheterization

## Abstract

This study aimed to assess the relationship between different LA strain components and PCWP as well as to the relationship with other established methods. We studied 144 symptomatic patients, age 63 ± 14 years, 54 males, using conventional transthoracic echocardiography protocols, including LA and LV myocardial deformation from speckle tracking technique investigations along with simultaneous right heart catheterization (RHC) using established techniques. From RHC, pulmonary artery pressure (PAP), and pulmonary capillary wedge pressure (PCWP) were measured and pulmonary vascular resistance (PVR) calculated. LA strain rate during atrial contraction (LASRa) was the strongest correlate with PCWP (r^2^ =  − 0.40, p < 0.001), over and above both LASR during LV systole (LASRs) and LA longitudinal strain during ventricular systole (LASs) (r^2^ = 0.21 and 0.19, respectively, p < 0.001 for both). The correlation between LASRa and PCWP was stronger in patients with post-capillary PH compared to pre-capillary PH (r^2^ = 0.21 vs. r^2^ = 0.02, respectively). The strongest relationship between LASRa and PCWP was in patients with enlarged LA volume > 34 ml/m^2^ (r^2^ = 0.60, p < 0.001). In all patients LASRa <  = 0.9 1/s was 88% accurate in predicting LA pressure > 15 mmHg which was superior to recently proposed uni- and multi-variable models. LASR during atrial contraction is the strongest predictor of PCWP, particularly in patients with post-capillary PH and with dilated LA cavity. Furthermore, it proved superior to recently proposed uni- and multi-variable based algorithms. Its close relationship with LV strain rate counterpart reflects important left heart chamber interaction in patients with raised LA pressure.

## Introduction

Left atrial pressure (LAP) changes is a corner stone explanation of symptoms in cardiac patients. In the absence of significant mitral valve disease, raised LAP is mainly due to left ventricular (LV) disease and raised diastolic pressures. Assessment of LAP has historically been an invasive investigation with catheter tip manometers placed in the peripheral pulmonary circulation, recording the pulmonary capillary wedge pressure (PCWP) as a reflection of LAP [1]. Echocardiography has become an essential tool for all cardiac patients irrespective of the underlying pathology. [2, 3] In those with suspected raised LV filling pressures, spectral Doppler is useful in demonstrating raised LAP which assist clinicians in prescribing LA pressure off-loading therapies [4]. However, Doppler parameters can sometimes be only modestly accurate in estimating LAP [5] even when combined with myocardial velocities. [6] To overcome single measurement limitation in estimating PCWP a multi-variable algorithm has been proposed in recent guidelines [3, 7].

Myocardial speckle strain LA function has recently developed [8]. Reduced peak longitudinal LA strain has been shown to correlate with raised LA pressures [9], despite potentially been influenced by a number of other co-factors. [10]

The aim of this study was to investigate the best accurate component of LA deformation function that predicts raised LA pressure and its relationship with its LV counterpart.

## Material and methods

We prospectively investigated 144 consecutive patients, which all underwent right heart catheterization (RHC) between 2010 and 2015. The cause for RHC were to assess the presence and severity of pulmonary hypertension as an explanation for patient’s breathlessness and potential diagnosis of heart failure. A previous study of a subgroup of such patients has been published. [11] A simultaneous Doppler echocardiographic examination including LV and LA deformation was performed and E/e’ lateral calculated. Normal RHC pressures were defined as mean pulmonary artery pressure (mPAP) < 20 mmHg. Pre-capillary pulmonary hypertension (pre-CPH) was defined as mPAP > 20 mmHg, PVR >  = 3WU and PCWP <  = 15 mmHg whereas post-capillary pulmonary hypertension (post-CPH) was defined as mPAP > 20 mmHg, PVR < 3WU and PCWP > 15 mmHg. A PH cut-off value of mPAP > 20 mmHg was used based on recent reports. [12]

### Right heart catheterization

Venous access was established by inserting a cannula in the right internal jugular vein, a medial cubital vein or in the right femoral vein. A retrograde catheterization was then performed using a Swan-Ganz® Standard Thermodilution Catheter (Edwards Lifesciences) [13]. Mean right atrial pressure (mRAP), systolic and end-diastolic right ventricular pressures, pulmonary artery systolic, mean and diastolic pressures (PASP, PAMP and PADP, respectively), and mean PCWP were all measured.

Blood samples for estimating oxygen saturation were drawn from the superior vena cava (SVC), pulmonary artery and femoral artery, 8% was considered a significant oxygen saturation step up between the SVC and the pulmonary artery. Cardiac output (CO) was determined by thermodilution [14] and pulmonary vascular resistance (PVR) was calculated using the equation PAMP − PCWP (trans-pulmonary gradient) divided by CO.

### Echocardiographic examination

Echocardiographic examination was performed using a Vivid E9 system (GE Medical Systems, Horten, Norway) equipped with an adult 1.5–4.3 MHz phased array transducer. Standard views from the parasternal long and short axis and apical four-chamber views were used. Flow velocities were obtained using pulsed and continuous wave Doppler techniques as proposed by the American Society of Echocardiography and European Association of Cardiovascular Imaging [15, 16]. All acquisitions were made from supine position because of the RHC procedure.

Stroke volume and CO measurements were made at the level of LV outflow tract. Trans-mitral blood flow velocities were measured with the sample volume placed at the tips of the mitral valve leaflets with optimal angulation to LV inflow. Pulmonary venous flow was measured from the apical four chamber view with the pulsed wave sample volume placed at the orifice of the vein. Retrograde systolic trans-tricuspid flow was obtained from either parasternal right ventricular inflow or apical 4-chamber view, for measuring peak retrograde trans-tricuspid pressure drop using continuous wave Doppler. Pulsed wave tissue Doppler recordings were also made to assess LV lateral myocardial early diastolic velocities (e’ lat) and E/e’ lateral was calculated. [17] All Doppler recordings were obtained at a sweep speed of 50–100 mm/s with a superimposed ECG (lead II). Off-line analysis was made using commercially available software (General Electric, EchoPac version BT 13, 113.0, Waukesha, Wisconsin, US) and means of three consecutive cardiac cycles were calculated. The study protocol was approved by the Regional Ethics Committee of Umeå (DNR 07–092 M) and all subjects gave an informed consent to participate in the study. Patients and the public were not (or will not) involved in study design, or conduct, or reporting, or dissemination plans of the study.

### Assessment of LA and LV deformation function

**Fig. 1:**
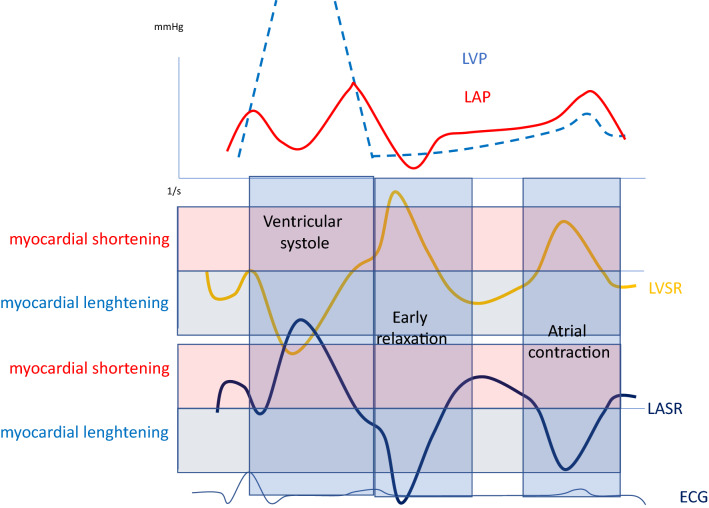
**a** Left atrial strain rate including deformation rate during ventricular systole (LASRs), early diastole (LASRe) and atrial contraction (LASRa). **b** Left atrial strain during ventricular systole (PALS). **c** Left ventricular strain rate including deformation rate during ventricular systole (LVSRs), early diastole (LVSRe) and atrial contraction (LVSRa). **d** Left ventricular strain during ventricular systole (LV GLS)

Anatomical landmarks were used and care was taken for echocardiographic image acquisition to ensure adequate LA tracking, avoiding foreshortening of LA cavity, interference with the pulmonary veins or LA appendage when measuring global strain and strain rate of LA and LV. Longitudinal myocardial deformation assessed by 2-dimensional echocardiography using speckle tracking was analyzed off-line. From the apical 4-chamber view, using a point-and-click technique creating a horseshoe shaped ROI within the LA and LV. The endocardial border of the septal, roof /apical and lateral wall of LA and LV were traced manually, in order to analyze global LA and LV strain and strain rate measurements, respectively, Fig. 1a–c. Tracings which didn't accurately track LA or LV structures were discarded.

Overall, at least 4 out of the 6 LA segments produced by the software was a necessary pre-requisite to include the patient in the study, and their average was taken for analysis [18]. Strain and strain rate recordings from 3 cardiac cycles were averaged, to assess the longitudinal LA deformation. The following parameters were calculated, with the reference point set at the onset of the QRS complex of the superimposed ECG. LASs (left atrial longitudinal strain during ventricular systole), LASRs (LA strain rate during ventricular systole), LASRe (LA strain rate during early ventricular filling phase) and LASRa (LA strain rate during late diastole/atrial systole) were all measured, Fig. 1.

Assessing longitudinal LV myocardial deformation was made from the apical 4-chamber view with the endocardial border carefully traced in the same fashion as described above. The following parameters were calculated, LV GLS (left ventricular global longitudinal strain), LVSRs (left ventricular strain rate during systole) LVSRe (LV strain rate during early ventricular filling phase), and LVSRa (left ventricular strain rate during late diastole), Fig. 1. Strain and strain rate analyses were measured using a dedicated work station (General Electric, EchoPac version BT 13, 113.0, Waukesha, Wisconsin, US).

All strain measures were expressed as positive values despite the fact that myocardial shortening being negative (LV systole and LA reservoir) in deformation and lengthening positive (LV diastole and LA conduit and contraction) in deformation.

### Statistics

The statistical software package IBM, SPSS (version 25) was used for all statistical tests and calculations. Patient characteristics and normally distributed data were expressed as mean ± standard deviation (SD). Correlations between parameters were tested using Pearson´s correlation coefficient and expressed as r^2^. ROC analysis was performed to assess prediction of abnormal PCWP (> = 15 mmHg). Sensitivity, specificity, positive (PPV) and negative (NPV) predictive values, as well as accuracy was calculated using different echocardiographic algorithms for elevated PCWP. A p-value < 0.05 was considered significant.

## Results

### Reproducibility of LA and LV speckle tracking measurements

The LA and LV strain and strain rate measurements inter-observer reproducibility has been extensively studied before. We had assessed LA strain and strain rate in a subgroup of the population and LASRa proved to have an inter-class correlation (ICC) of 0.89, p < 0.001. [11] Other researchers found a similar ICC for LA strain and strain rate inter-observer reproducibility (ICC = 0.74–0.86, p < 0.01) [19] as well as LV strain with ICC > 0.89 [20].

### Patients clinical, hemodynamic and echocardiography characteristics (Tables 1 and 2)

**Table 1:** Clinical characteristics

Diagnosis	Number
ASD/VSD	5
Rheumatic disease (SSc,SLE,RA,MCD)	30 (24,2,1,3)
CTPH	12
Pulmonary disease	13
Heart failure with LVEF > = 50% (%)	63
Heart failure reduced LVEF, < 50% (%)	37
Other (sarcoidosis, constrictive pericarditis, cardiac amyloidosis)	4
Systemic hypertension	55
Diabetes mellitus	17
Ischemic disease	22
Medications	
Betablockers	56
ACE/ARB	78
Diuretics	84
PAH medication	46

*ASD* atrial septum defect, *SSc* systemic sclerosis, *SLE* systemic lupus erythematosus, *RA* reumatiod arthritis, *MCD* mixed connective disease, *LVEF* left ventricular ejection fraction

**Table 2:** Clinical demographics, RHC and echocardiographic results

	N	Mean	SD
Height, cm	144	169	10
Weight, kg	144	78	17
BSA, m^2^	144	2.0	0.2
SBP, mmHg	144	132	20
DBP,mmHg	144	78	11
NT-proBNP, ug/L	121	1893	3155
Log NT-proBNP,ug/L	121	2.8	0.6
Right heart catheterization			
TPG,mmHg	142	20	14
PVR,WU	143	4.2	3.3
RAP,mmHg	143	8.3	5.6
PCWP,mmHg	144	12.8	6.2
RVEDP,mmHg	115	10.5	5.1
MPAP,mmHg	144	33	14
Echocardiography			
IVS,mm	129	10.7	2.7
LVDD,mm	130	49	10
PWT,mm	125	8.1	2.1
HR,bpm	144	74	14
IVRT,ms	118	86	28
DT,ms	131	152	58
Lat s’,cm/s	125	6.5	2.1
Lat e’,cm/s	125	8.2	3.4
LVEF,%	135	53	13
E/e’ lateral	111	10.7	5.9
PVF S/D	67	1.0	0.5
LAVI,ml/m^2^	134	33	19
TR gradient, m/s	125	47	19
LA strain/strain rate			
LASRs, 1/s	138	0.84	0.45
LASRe, 1/s	134	0.82	0.57
LASRa, 1/s	108	1.21	0.64
LASs, %	138	15.1	9.0
LV strain/strain rate			
LVSRs, 1/s	132	0.81	0.29
LVSRe, 1/s	133	1.01	0.53
LVSRa, 1/s	108	0.83	0.43
LV GLS, %	136	14.5	6.1

*BSA* body surface are, *TPG* transpulmonary gradient, *PVR* pulmonary vascular resistance, *RAP* right atrial pressure, *PCWP* pulmonary capillary pressure, *RVEDP* right ventricular end diastolic pressure, *MPAP* mean pulmonary artery pressures, *IVS* interventricular septum, *PWT* posterior wall thickness, *LV* left ventricular, *DD* diastolic diameter, *SD* systolic diameter, *IVRT* isovolumic relaxation time, *DT* deceleration time, *Lat* lateral wall, *s*’ systolic myocardial velocity, *e*’ early diastolic velocity, *LAVI* left atrial volume indexed for BSA, *TR* tricuspid regurgitation, *LA* left atrial, *SR* strain rate, *s* systole, *e* early diastole, *a* atrial, *LV* left ventricular, *GLS* global longitudinal strain, *LASs* atrial longitudinal strain during ventricular systole

Patients had a variety of clinical diagnoses. From RHC 15% had normal PA pressures, 47% had pre-capillary PH, 12% had post-capillary PH and 26% had combined pre and post-capillary PH. Sixty two percent of patients had preserved LV ejection fraction (EF > 50%) and the rest had reduced EF (≤50%). Approximately 39% of the total number of patients had hypertension but only 15% had coronary artery disease. No patient had significant (> mild) aortic or mitral valve disease. Four patients had moderate TR. 37% had LA volume > 34 ml/m^2^. Most patients were taking ACE-I, A_2_-blockers or diuretics, the rest were on beta-blockers or pulmonary hypertension medications (Table 1). 90/144 studied patients were females and 54 were males. 33 patients had atrial fibrillation.

### Conventional echocardiographic measures and their relationship with LV filling pressures

**Fig. 2:**
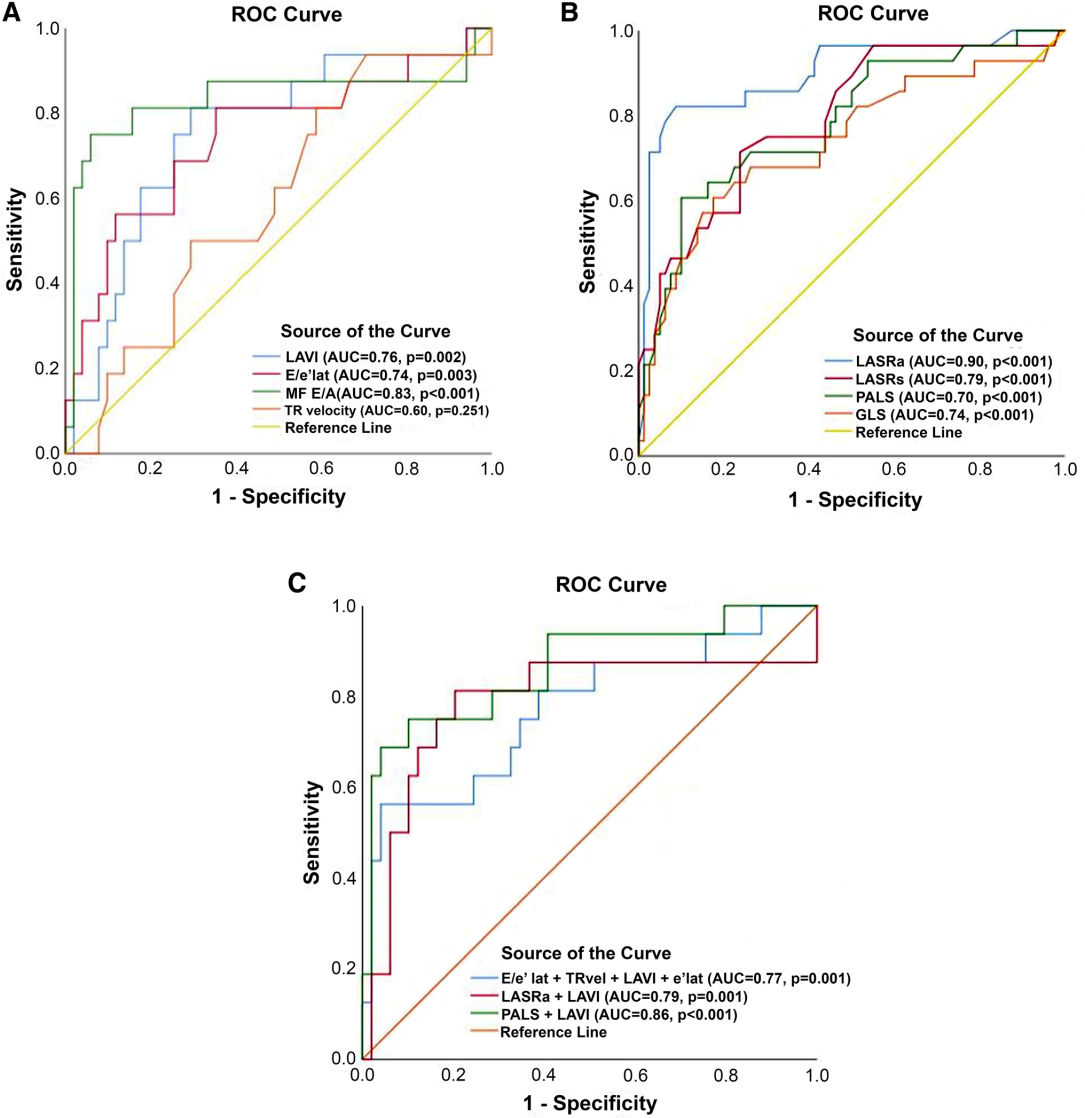
**a** ROC analysis testing uni-variable model (E/e’, TR vel, LAVI and E/A) in predicting elevated PCWP. **b** ROC analysis testing uni-variable LA and LV strain and strain rate model in predicting elevated PCWP. **C** ROC analysis testing multivariable models in predicting elevated PCWP

Mitral E/A had the highest r^2^-value (r^2^ = 0.41) in relating to PCWP, followed by pulmonary venous flow ratio of systolic and diastolic flow velocities (r^2^ = 0.32), mitral DT (r^2^ = 0.25), E/e’ lateral (r^2^ = 0.18) and IVRT (r^2^ = 0.16), Table 3. E/A relationship with PCWP increased and became highly significant (r^2^ = 0.50) after including patients with large LA volume (> 34 ml/m^2^). E/A proved to have the strongest relationship with abnormal PCWP (AUC = 0.83, p < 0.001), Fig. 2a.

**Table 3:** Correlations between conventional echocardiography, LA and LV deformation and PCWP in all patients and in patients with increased LA volume

All patients				Patients with LAVI > 34 ml/m^2^		
	n	r^2^	p-value	N	r^2^	p-value
LA strain						
LASRs,1/s	137	0.21	< 0.001	48	0.14	0.010
LASRe,1/s	133	0.03	0.061	47	0.00	0.814
LASRa,1/s	108	0.40	< 0.001	32	0.60	< 0.001
LASs,%	138	0.19	< 0.001	48	0.17	0.004
LV strain						
LVSRs,1/s	131	0.14	< 0.001	45	0.10	0.041
LVSRe,1/s	132	0.27	0.1	45	0.01	0.215
LVSRa,1/s	132	0.27	< 0.001	32	0.32	0.001
LV GLS,%	136	0.13	< 0.001	47	0.16	0.006
Conventional Doppler						
Mitral DT	131	0.25	< 0.001	43	0.17	0.03
IVRT	118	0.16	< 0.001	38	0.18	0.07
E/A	108	0.41	< 0.001	30	0.50	< 0.001
E/e’ lat	95	0.18	< 0.001	33	0.07	0.133
PVF, S/D	67	0.32	< 0.001	20	0.34	0.05

*LA* left atrial, *SR* strain rate, i systole, *e* early diastole, *a* atrial, *LV* left ventricular, *GLS* global longitudinal strain, *PALS* peak atrial longitudinal strain

### LA & LV deformation and PCWP

LA deformation function in all patients showed LASRs and LASs correlating weakly with PCWP (r^2^ = 0.21 and r^2^ = 0.19, respectively, p < 0.001 for both) and LASRe not correlating with PCWP (r^2^ = −0.03). However, LASRa correlated modestly with PCWP (r^2^ = 0.40, p < 0.001), Table 3. The same correlation strength was found between LV deformation parameters and PCWP, at each phase of the cardiac cycle, Table 3.

**Fig. 3:**
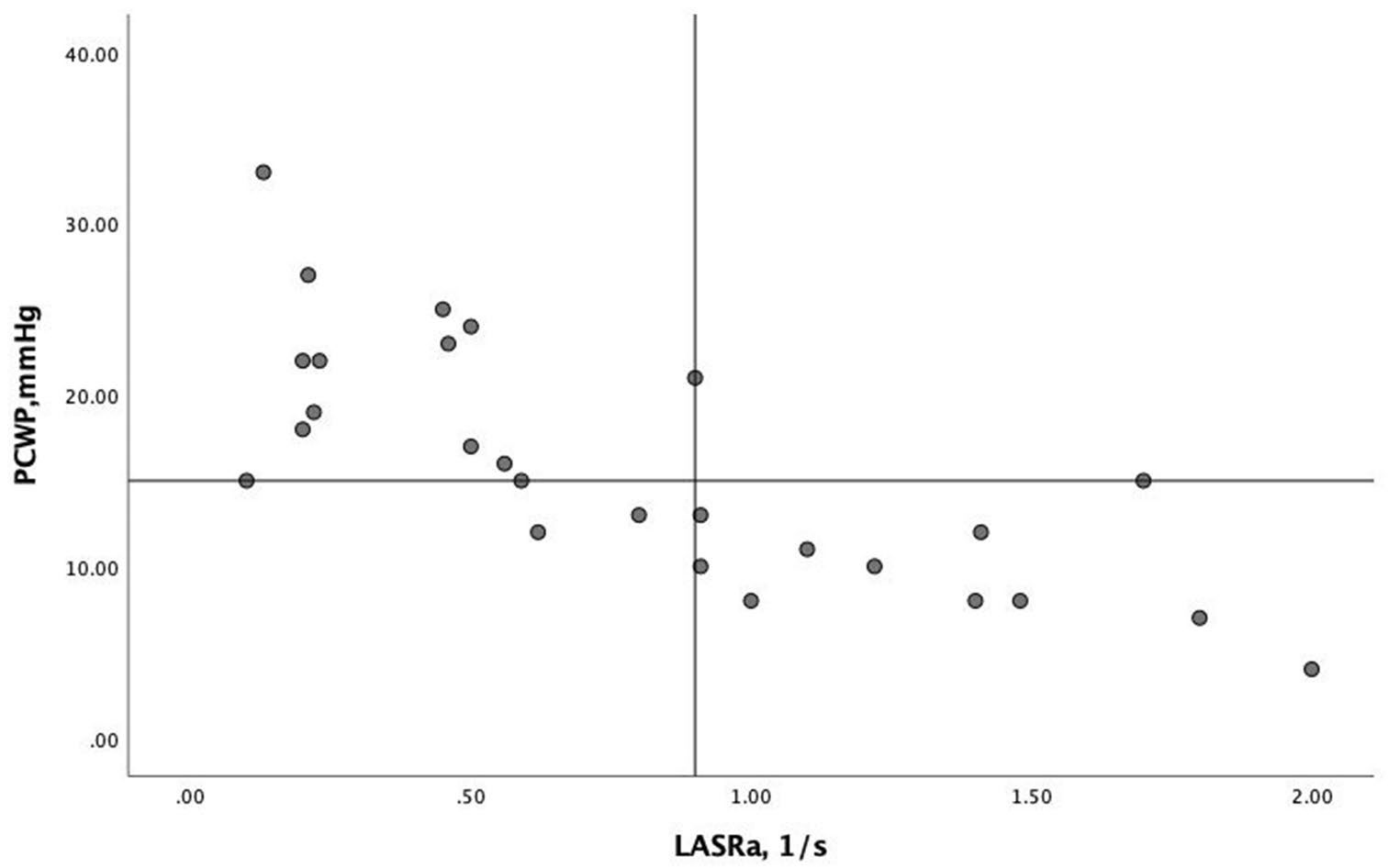
LASRa and PCWP in patients with LA volume > 34 ml/m^2^ Vertical line 0.9 1/s and horizontal line 15 mmHg

The relationship between LASRa and PCWP was stronger in patients with post-CPH compared to those with pre-CPH and combined CPH (r^2^ = 0.21 vs. r^2^ = 0.02 and r^2^ = 0.08). The strongest relationship between LASRa and PCWP was found in patients with LA volume > 34 ml/m^2^ (r^2^ = 0.59, p < 0.001, Fig. 3), an observation that was not found in any of the other strain measures. ROC analysis showed LASRa to have the largest AUC (0.90, p < 0.001) in predicting PCWP >  = 15 mmHg with a LASRa < 0.90 1/s having a sensitivity of 82% and a specificity of 91%. Figure 2b.

Among all strain and strain rate measurements as well as conventional echo measurements in patients with increased LA volume, LASRa was still the most accurate deformation measurement that predicted raised LA pressure, Table 3. LASRa <  = 0.9 1/s had 100% sensitivity, 73% specificity, 100% negative predictive value and 75% positive predictive value with 85% accuracy in predicting PCWP > 15 mmHg**.**

### Correlation between LA and LV deformation (supplemental file, Table 5)

LA and LV strain and strain rate measurements at each phase of cardiac cycle, systole, early diastole and atrial systole, correlated either weakly (r^2^ = 0.07, p = 0.004 for early diastole) or only modestly (ventricular and atrial systole, r^2^ = 0.18 and 0.28, p < 0.001) with each other.

### Recommended multi- and univariate models for predicting elevated PCWP

To test multi-variable approach for identifying raised PCWP we evaluated an algorithm previously proposed in patients with both normal and reduced LVEF [7]. This algorithm used e’ < 10 cm/s, (lateral segment) E/e’ lateral > 12, TR velocity > 2.8 m/s and LAVI > 34 ml/m^2^. The presence of >  = 3 criteria suggested elevated PCWP. We also tested the combination of LASRa and LAVI as well as PALS and LAVI as predictors of elevated PCWP. We found the algorithm from Oh et al. having AUC of 0.77, p = 0.001. The predictive ability was slightly better using LASRa + LAVI (AUC = 0.79, p = 0.001) and PALS + LAVI (AUC = 0.86, p < 0.001), Fig. 2c. Testing sensitivity, specificity, positive and negative predictive values as well as accuracy in all patients showed that LASRa had the highest accuracy (88%) followed by E/e’ lateral (74%), multi-variable model (70%) and TR velocity (42%), Table 4.

**Table 4:** Prediction of elevated PCWP using modified multi variable model and uni-variable models

Model	Sensitivity	Specificity	PPV	NPV	Accuracy
Modified algorithm, combined model [7]	42	80	42	80	70
E/e’ lateral > 12	47	86	61	78	74
TR velocity > 2.8 m/s	80	28	40	70	48
LASRa, < 0.9 1/s	70	97	92	86	88

## Discussion

### Findings

The findings of this study are summarized as follows: (1) Among the LA strain and strain rate measurements, strain rate during atrial contraction was the best accurate component of LA deformation measurements that correlated with PCWP; (2) Such relationship was particularly strong in patients with post-CPH and with enlarged LA, volume > 34 ml/m^2^.

(3) In patients with large LA volume, LASRa <  = 0.9 1/s was 85% accurate in predicting LA pressure > 15 mmHg; (4) The strongest relationship between LA and LV deformation measurements were between SR during atrial contraction, the remaining were only modest. (5) The LASRa had superior accuracy in predicting elevated PCWP compared to recently proposed uni- and multi-variable based algorithms.

### Data interpretation

The findings of this study were not met with a surprise since the components of the strongest relationship, we found, between LA structure and function and PCWP represent a continuum of blood flow and pressure. The LA and the pulmonary veins and venules are the closest part of the pulmonary circulation to the distal territory of the pulmonary arterial capillary where the PCWP is conventionally measured. Thus, irrespective of the cause any rise in LA pressure is expected to be reflected on the pulmonary venous circulation pressure, and subsequently on the PCWP. A number of factors impact the extent of such pressure transfer, the most important is the intrinsic properties of the LA myocardium. A slow rise of LA pressure in a normal myocardium could be smoothly accommodated by cavity dilatation, without significant impact on the pulmonary venous circulation or pressures. On the other hand, perpetual longstanding rise of LA pressure causes chamber enlargement, myocardial stretch and significant rise of cavity stiffness [21, 22]

LA volume, and indices of LA stretch and stiffness have been shown to correlate with severity of LA pressure rise, symptoms and clinical outcome. [23] Also reduced LA strain and strain rate during both reservoir and contraction phases have been shown to improve after decongestive therapy in HFrEF patients [24].

LA myocardial function assessed by 2D strain analysis is much more complex than that of the thicker LV, for which the current available softwares are produced. Such complexity is based on the assessment of cavity strain and strain rate during the three cardiac phases; LV systole, LV early diastole and during LA systole, thus creating six variables. While the LA deformation measurements during LV systole (LASs) has commonly been taken as the best reflector of LA cavity deformation and stretch [8, 25], our findings show that LA deformation during atrial contraction is the strongest predictor of raised LA pressure, particularly in patients with raised LA pressure and enlarged cavity. By applying Frank Starling law, the LA cavity enlargement is associated with myocardial thinning, suppressed contractile function and hence compromised deformation, which our results support. The cause for better relationship between LASRa and PCWP in post-capillary PH is probably due to the non-linear relationship between LASRa and PCWP in the whole group. In adhering with previous studies and meta-analyses that reported LASs as the strongest predictor of cavity pressure and recurrent arrhythmia [26, 27], the compromised LA strain rate during atrial contraction, we found, could be seen as directly reflecting LA myocardial dysfunction. This finding should indeed be considered in a fashion parallel to the left ventricle.

As LASs, is to a great extent, influenced by LV deformation, LA strain rate during atrial contraction, in our patients, seem also to be influenced by its LV counterpart. Atrial contraction is an important phase of the atrial function and is the first to face any rise of LV end-diastolic pressure, in patients with LV disease, which results in significant prolonged time flow reversal in the pulmonary veins [28]. Our strong correlation between the strain rate of the two chambers, LA and LV, during end-diastole supports an important evidence for LA-LV function interaction in patients with raised LA pressure. These findings highlight a spring like relationship between LA and LV with contraction of one requiring relaxation of the other, during the same phase of the cardiac cycle. The weak atrio-ventricular relationship of the early relaxation phase might reflect the complexity of mechanical ventricular and atrial relaxation.

### Clinical implications

LA strain rate during atrial contraction seems to be the most accurate deformation measurement, that inversely correlates with PCWP, over and above the recently proposed multi-variable algorithm. The accuracy of such measurement is significantly enhanced in patients with post-capillary PH and with enlarged LA size, volume > 34 ml/m^2^. These patients are the commonly seen in cardiology clinics who present with exertional breathlessness and frequent atrial arrhythmias. Most patients with such findings have long standing hypertension, with or without additional pathologies e.g. coronary artery disease, thus highlighting the invaluable role of LASRa measurements in explaining their symptoms, particularly those with no or hardly measurable tricuspid regurgitation as an estimate for systolic pulmonary artery pressure. Finally, for practical daily application, in patients with increased LA volume, a reduced LASRa <  = 0.9 1/s was 85% accurate in predicting raised LA pressure > 15 mmHg.

### Limitations

PCWP measurements were made using conventional protocols which are known for their potential technical variability [29]. The strain measures were only assessed from the four-chamber view which is a limitation when some of the guidelines recommend a bi plane approach. These might need further discussion when using strain or strain rate as the two are technically related to each other. However, we found strain rate being easier to use in measuring the diastolic phase of atrio-ventricular function. However, as patients were in supine position and studied during invasive procedure, the acquisition feasibility was limited. Testing e’ as indirect measure of PCWP was only measured at the lateral wall, since 47% of patients had pre capillary pulmonary hypertension so we did not consider septal e’ reliable in assessing E/e’ as indirect measure of PCWP. [17] It might be argued if an abnormal PCWP should be more than 12 or 15 mmHg. We have considered a cut off of 15 mmHg because it is the most used and has proved accurate in clinical practice.

### Conclusions

LASR during atrial contraction is the strongest predictor of PCWP, particularly in patients with post-capillary PH and dilated LA cavity. Its close relationship with LV strain rate counterpart reflects important left heart chamber interaction in patients with raised LA pressure.
